# An immune‐related prognostic signature for predicting breast cancer recurrence

**DOI:** 10.1002/cam4.3408

**Published:** 2020-08-25

**Authors:** Zelin Tian, Jianing Tang, Xing Liao, Qian Yang, Yumin Wu, Gaosong Wu

**Affiliations:** ^1^ Department of Thyroid and Breast Surgery Zhongnan Hospital of Wuhan University Wuhan China

**Keywords:** breast cancer, DEGs, IRGs, prognostic signature, transcription factor

## Abstract

Breast cancer (BC) is the most common cancer among women worldwide and is the second leading cause of cancer‐related deaths in women. Increasing evidence has validated the vital role of the immune system in BC development and recurrence. In this study, we identified an immune‐related prognostic signature of BRCA that could help delineate risk scores of poor outcome for each patient. This prognostic signature comprised information on five danger genes—*TSLP, BIRC5, S100B, MDK*, and *S100P*—and three protect genes *RARRES3, BLNK*, and *ACO1*. Kaplan‐Meier survival curve showed that patients classified as low‐risk according to optimum cut‐off risk score had better prognosis than those identified within the high‐risk group. ROC analysis indicated that the identified prognostic signature had excellent diagnostic efficiency for predicting 3‐ and 5‐years relapse‐free survival (RFS). Multivariate Cox regression analysis proved that the prognostic signature is independent of other clinical parameters. Stratification analysis demonstrated that the prognostic signature can be used to predict the RFS of BC patients within the same clinical subgroup. We also developed a nomogram to predict the RFS of patients. The calibration plots exhibited outstanding performance. The validation sets (GSE21653, GSE20711, and GSE88770) were used to external validation. More convincingly, the real time RT‐PCR results of clinical samples demonstrated that danger genes were significantly upregulated in BC samples, whereas protect genes were downregulated. In conclusion, we developed and validated an immune‐related prognostic signature, which exhibited excellent diagnostic efficiency in predicting the recurrence of BC, and will help to make personalized treatment decisions for patients at different risk score.

## Graphical abstract

This study developed and validated an immune‐related prognostic signature, which exhibited excellent diagnostic efficiency in predicting the recurrence of BC, and will help to make personalized treatment decisions for patients at different risk score.

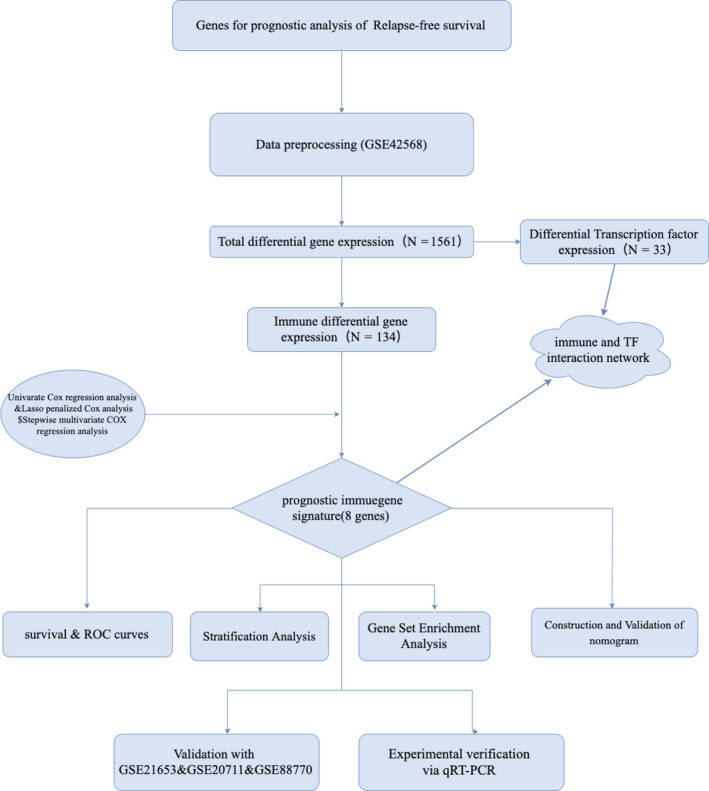

## 1 INTRODUCTION

BRCA is the most commonly diagnosed cancer in women. The 2018 GLOBOCAN report revealed that approximately 2.1 million women worldwide were diagnosed with BC in 2018, accounting for one‐fourth of all cancer cases among women.^1^ In recent decades, advances in medical technology have contribute for the gradual reduction of BC‐associated mortality; however, the prognosis of relapsed patients remains poor.^2^, ^3^ BC is a heterogeneous malignancy that affects a diverse population,^4^ for which treatment strategies often include systemic and personalized therapies.^4^, ^5^ Due to different clinic‐pathological features, BC patients manifest broad range of treatment‐related side‐effects and clinical outcomes.^6^ Therefore, new alternative diagnostic methods are urgently needed to identify high‐risk BC patients and provide guidance for the optimization of personalized treatments.

Increasing evidence has shown that the immune system plays a critical role in BC recurrence.^7^, ^8^, ^9^ Recent discoveries on tumoral immune evasion have paved the way for increasing interest in cancer immunology.^10^, ^11^ Cancer immunoediting is a dynamic process consisting of three main stages— elimination, equilibrium, and evasion—ultimately aiming to destroy cancer cells.^12^, ^13^ During the immune evasion stage, cancer cells use various mechanisms to evade attack and resist the immune response of the host, while activating pro‐survival and pro‐proliferation processes.^14^ Common tumor‐associated immune evasion mechanisms include abnormal expression of tumor‐associated antigens (TAAs), loss or modification of major histocompatibility complex class I (MHC‐1), and activation of anti‐apoptotic mechanisms.^11^ Immunosuppressant drugs targeting PD‐L1/PD‐1 and CTLA‐4 were designed to prevent some of these evasion mechanisms, effectively promoting cancer regression and improving patient prognosis.^15^, ^16^, ^17^, ^18^

In recent years, "omics" technology has developed rapidly. Microarray analysis and whole‐genome sequencing have granted the possibility to explore the genomic characteristics of high‐risk cancers.^19^, ^20^ In particular, genomics and molecular characterization studies have revealed driving mechanisms of BC.^20^, ^21^ Several studies have shown that multi‐gene signature models based on the analysis of tumor arrays can help predict cancer prognosis and recurrence more accurately than conventional methods.^22^, ^23^, ^24^ However, the immune‐related genes (IRGs) prognosis signature is rarely described. In this study, we analyzed IRGs from Gene Expression Omnibus (GEO) dataset and developed an immune‐related prognostic signature, providing novel insights for identifying high‐risk BC and assessing the potential of personalized immunotherapy for treating BC patients.

## 2 MATERIALS AND METHODS

### 2.1 Training and validation datasets

IRGs expression data and clinical information were obtained from the GEO database. GSE42568 dataset was used as the training set, while GSE21653, GSE20711, and GSE88770 were used for validation. GPL570 [HG‐U133_Plus_2] Affymetrix Human Genome U133 Plus 2.0 Array was used for gene annotation. We downloaded the original expression profile, and used the robust multi‐array average (RMA) algorithm to perform background correction and quantile normalization. Briefly, for multiple probes corresponding to one gene symbol an average value was considered; single probes corresponding to multiple gene symbols were deleted. After removing incomplete prognostic cases, a total of 121 cases were included in the training set (normal = 17; cancer = 104) and 435 cases were included in the validation set (237 in GSE21653; 85 in GSE20711, and 113 in GSE88770). Clinical information, such as T‐stage, ER status, lymph nodes, grade, and recurrence‐free survival were extracted from the dataset for further analysis.

### 2.2 Identification of differentially expressed genes

We used the *t* test method to evaluate the *P*‐value of each gene to determine whether the gene is differentially expressed in tumor and adjacent samples. The Benjamini‐Hochberg method was used to calculate the False Discovery Rate (FDR) to prevent false positive probability in multiple comparisons. Fold change (FC) was used to represent the ratio of gene expression levels between tumor and adjacent samples. Considering FDR <0.05 and | logFC |> 1 as inclusion criteria, the “limma” R package was used to screen differentially expressed genes (DEGs) in the training set. Download all immune genes through the ImmPort website online, and select the immune genes from DEGs(https://www.immport.org/shared/genelists). Then download tumor‐related transcription factors from the Cistrome Cancer website and screen out differentially expressed transcription factors (http://cistrome.org/Cistrome Cancer/).

### 2.3 Immune‐related prognostic signature construction and validation

Univariate Cox proportional hazard regression model was used to screen IRGs associated with RFS in the training set. *P*‐value < .05 was used as the inclusion criteria to identify candidate IRGs. Hazard ratio (HR) was used to identify IRGs into protect or danger, with HR <1 being considered as protect genes and HR >1 as danger genes. Lasso penalized COX analysis and stepwise multivariate Cox analysis were used to further narrow down the list of significant IRGs. Finally, we developed an immune‐related prognostic signatures (8 genes) based on the following risk score formula:

$$\text{Risk score}=\sum_{i=1}^{n}\exp_{i}^{*}\beta_{i}$$

where n represents the number of prognostic genes, expi is the expression value of gene i, and β_i_ is the univariate Cox regression coefficient of gene i. The patients were classified as high‐ or low‐risk according to the optimum cut‐off risk score. We used GSE21653, GSE20711, and GSE88770 to validate the accuracy of the identified immune‐related prognostic signature.

### 2.4 Construction of the nomogram

In this study, we used "rms" R package to construct a nomogram including clinical information (T_stage, nodes, ER status, and grade) and immune‐related signature in GSE42568, GSE21653, and GSE20711 datasets. GSE88770 was not included in further analysis due to lack of clinical information (T_stage). Calibration plots were used to evaluate the diagnostic efficiency of nomogram.

### 2.5 Acquisition of human BRCA samples

BRCA and paired adjacent tissue samples were taken from patients undergoing breast surgery at Zhongnan Hospital of Wuhan University. All specimens were collected after obtaining informed consent by the patients. The samples were immediately stored in liquid nitrogen for further experiments. The Ethics Committee of Zhongnan Hospital (Wuhan, Hubei) approved the use of these samples for total RNA isolation and quantitative reverse transcription‐polymerase chain reaction (qRT‐PCR) analysis. This study included 40 pairs of samples.

### 2.6 Total RNA extraction and qPCR analysis

RNeasy plus mini kits (74134, Qiagen) and 2 × SYBR Master Mix (TOYOBO, Japan) were used to extract total RNA according to the protocol provided by the manufacturer. qRT‐PCR was conducted in triplicate. GAPDH was used as internal control, and the 2^−ΔΔCt^ values were normalized to its levels. The primer sequences for qPCR used in this study are shown in Supplementary Table 1.

**Table 1:** Univariate and multivariate Cox regression analyses were performed on the gene signatures and RFS of BC patients

Variables		Patients(N)	Univariate analysis		Multivariate analysis	
			HR(95% CI)	*P*	HR(95% CI)	*P*
GSE42568						
T_stage	I/II&III	18/83	1.29(0.57‐2.89)	0.538	0.94(0.37‐1.96)	0.69
ER	∓	34/67	0.44(0.24‐0.79)	6.35e‐03	0.38(0.20‐0.74)	4.19e‐03
Nodes	∓	44/57	4.55(2.19‐9.48)	5.20e‐05	4.82(2.23‐10.39)	6.11e‐05
Grade	I&II/III	50/51	2.82(1.51‐5.29)	1.18e‐03	1.05(0.50‐2.18)	0.90
Risk score	Low/High	50/51	4.02(2.07‐7.83)	4.14e‐05	3.60(1.72‐7.52)	6.54e‐04
Total（GSE42568&GSE20711&GSE21653）						
T_stage	I/II	119/239	1.18(0.82‐1.71)	0.38	1.08(0.74‐1.59)	0.68
T_stage	I/III	119/65	2.29(1.39‐3.76)	1.06e‐03	1.86(1.12‐3.11)	1.58e‐03
ER	∓	179/244	0.61(0.45‐0.84)	2.41e‐03	0.67(0.47‐0.94)	2.14e‐03
Nodes	∓	186/237	2.14(1.51‐3.02)	1.68e‐05	1.84(1.29‐2.64)	8.22e‐04
Grade	I/II	66/126	1.86(1.00‐3.47)	4.99e‐03	1.73(0.91‐3.26)	0.09
Grade	I/III	66/231	2.59(1.45‐4.62)	1.27e‐03	1.73(0.94‐3.20)	0.08
Risk score	Low/High	210/213	2.53(1.80‐3.56)	8.49e‐08	2.32(1.64‐3.29)	1.87e‐06

### 2.7 Gene set enrichment analysis

The GSEA software (GSEA version 4.0.3) was used to develpoed a gene set enrichment analysis (GSEA) in training and validation sets. The samples were divided into high‐risk and low‐risk groups based on the cut‐off risk score. The c2.cp.kegg.v6.2.symbols.gmt gene set was chosen as the reference gene set. FDR is the adjusted P‐value after multiple hypothesis testing, FDR <25% (Benjamini‐Hochberg) was used as cutoff for significant gene sets. The most significant Kyoto Encyclopedia of Genes and Genomes (KEGG) pathways were screened.

### 2.8 Statistical analysis

In this study, we used Kaplan‐Meier plots to evaluate the differences in patients with low‐risk and high‐risk group, and used log‐rank tests to assess the statistical significance. *P*‐value < .05 (log‐rank tests) was considered statistically significant. Multivariate Cox regression analysis and stratification analysis were used to determine whether the immune‐related signature was an independent prognostic factor. The "survivalROC" R package was used for time‐dependent receiver operating characteristic (ROC) analysis, and the prognostic performance was verified by comparing the area under the ROC curve (AUC). All statistical tests were performed using R software (version 3.6.1).

## 3 RESULT

### 3.1 Identification of differentially expressed genes

We used GEO BC dataset GSE42568 as training set, which included 17 normal samples and 104 tumor samples. Using FDR <0.05 (Benjamini‐Hochberg) and $\left|\log\text{FC}\right|>1$ inclusion criteria, we identified 1561 DEGs, among which 134 were immune‐related genes and 33 were genes coding for TFs. Volcano plot and heatmap of the training set were shown in Supplementary Figure 1. Based on univariate Cox regression analysis, lasso regression analysis, and stepwise multivariate analyses, we developed an immune‐related prognosis signature (eight genes). The interaction network between the immune‐related signature and TFs was shown in Figure 2C.

**Figure 1:**
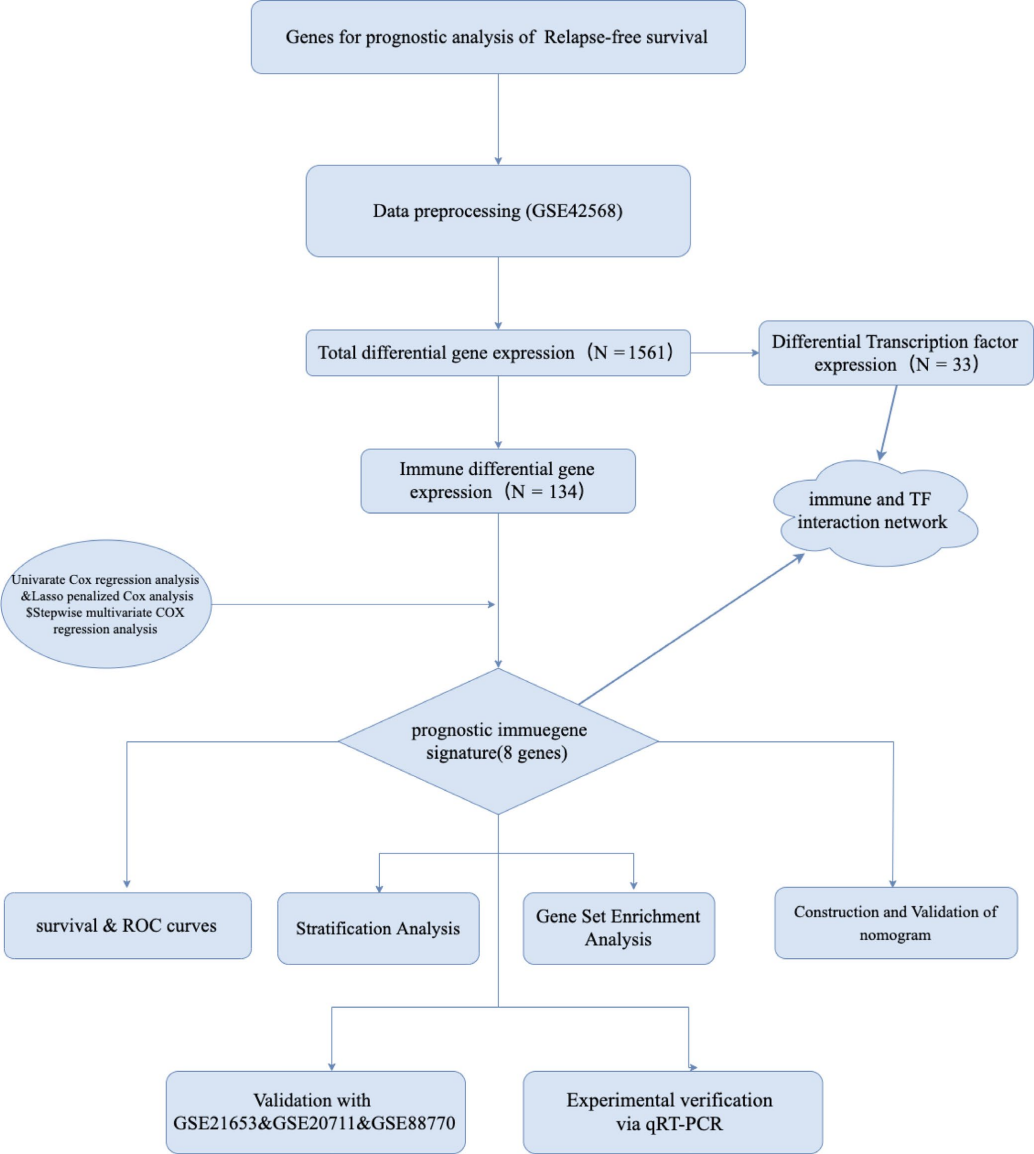
Flow diagram of data preparation, processing, analysis, and validation

**Figure 2:**
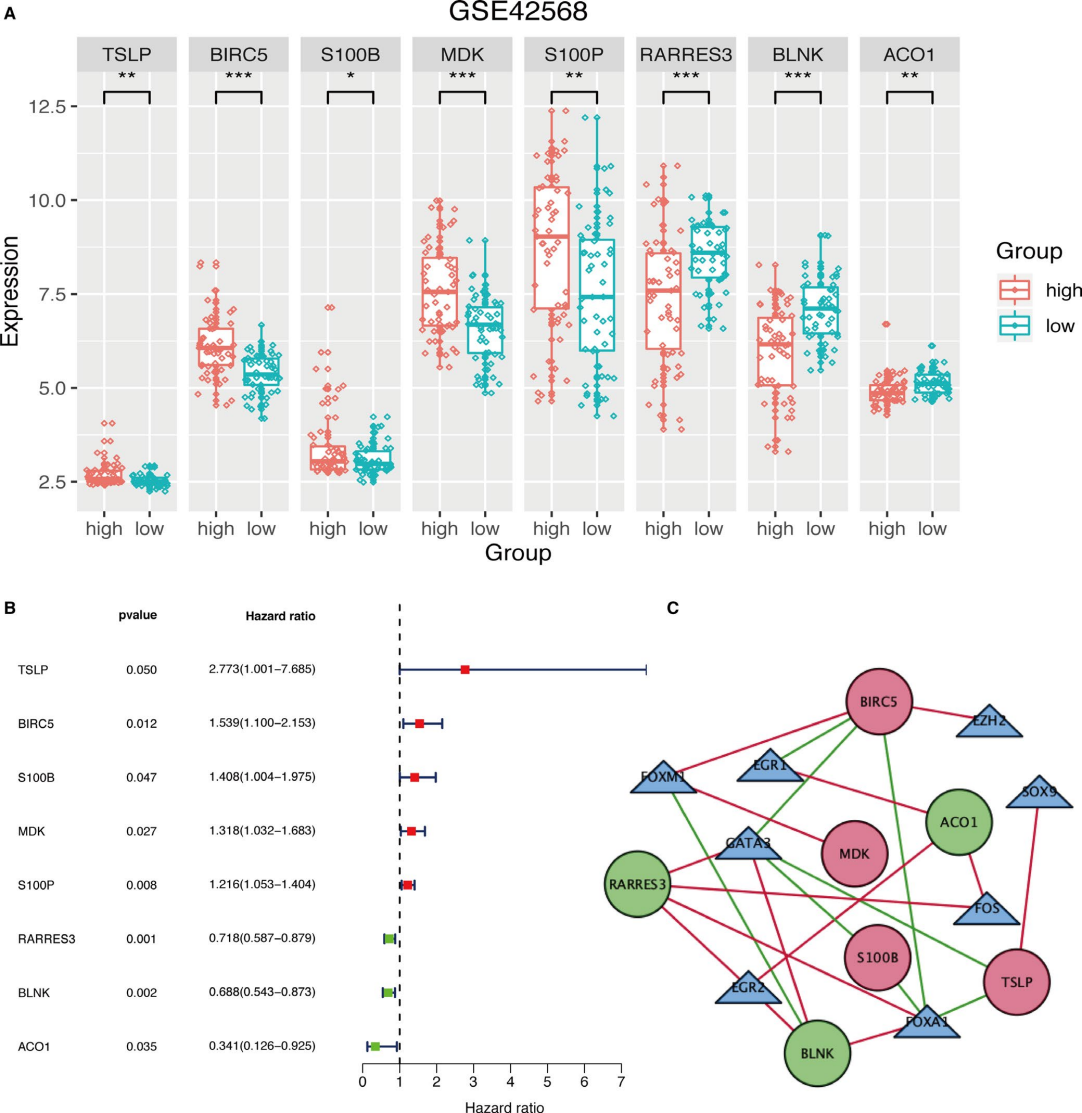
Immune‐related prognostic signature in training set. A, Expression levels of IRGs of the prognostic signature between low‐ and high‐group in training set. *P*‐value < .05 (*t* test) was considered statistically significant. *P < .05; **P < .01; ***P < .001. B, Univariate Cox regression analysis on the RFS of prognostic signature. C, Interaction network of IRGs and TFs. Red circles represent danger IRGs, green circles represent protective IRGs, and triangles represent TFs. The red line represents a positive correlation, and the green line represents a negative correlation.

### 3.2 Immune‐related prognostic signature generation

**Figure 3:**
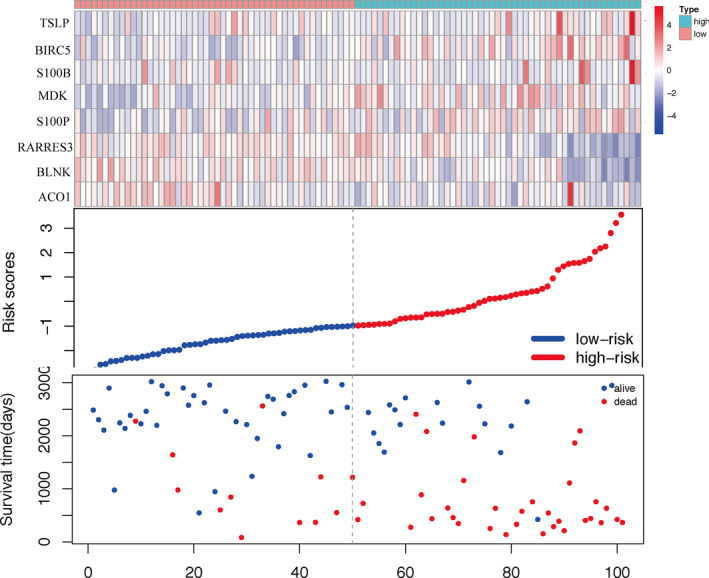
Analysis of risk score for BC patients in training set, with gene expression profile (top), risk score distribution (middle), and patient survival status (bottom). The black dashed line represents the cut‐off of the risk score, which divides patients into high‐risk and low‐risk groups

We first performed a univariate Cox regression analysis to identify RFS‐related prognostic genes in training set. According to the optimal cut‐off risk score, patients were divided into two groups: high‐risk and low‐risk. We identified 19 immune‐related prognostic genes, which were further evaluated by lasso‐penalized Cox regression and stepwise multivariate analysis (Supplementary Figure 2A‐B). This approach allowed us to identify five danger genes—*TSLP*, *BIRC5*, *S100B*, *MDK*, and *S100P*—and three protective genes—*RARRES3*, *BLNK*, and *ACO1*. This short list of eight genes was used to develop an immune‐related gene prognostic signature. Figure 2A indicated the expression of the immune‐related signature in training set. The results revealed that high‐risk group patients had higher expression levels of danger genes while low‐risk group patients had higher expression levels of protect genes. Figure 2B described hazard ratio of the immune‐related signature. In Figure 3, the ranking was based on the risk score values of the immune‐related signatures from low to high, the risk score distribution, risk gene expression and patient survival status in training set are shown, respectively.

### 3.3 Analysis of the immune‐related signature in the training set

**Figure 4:**
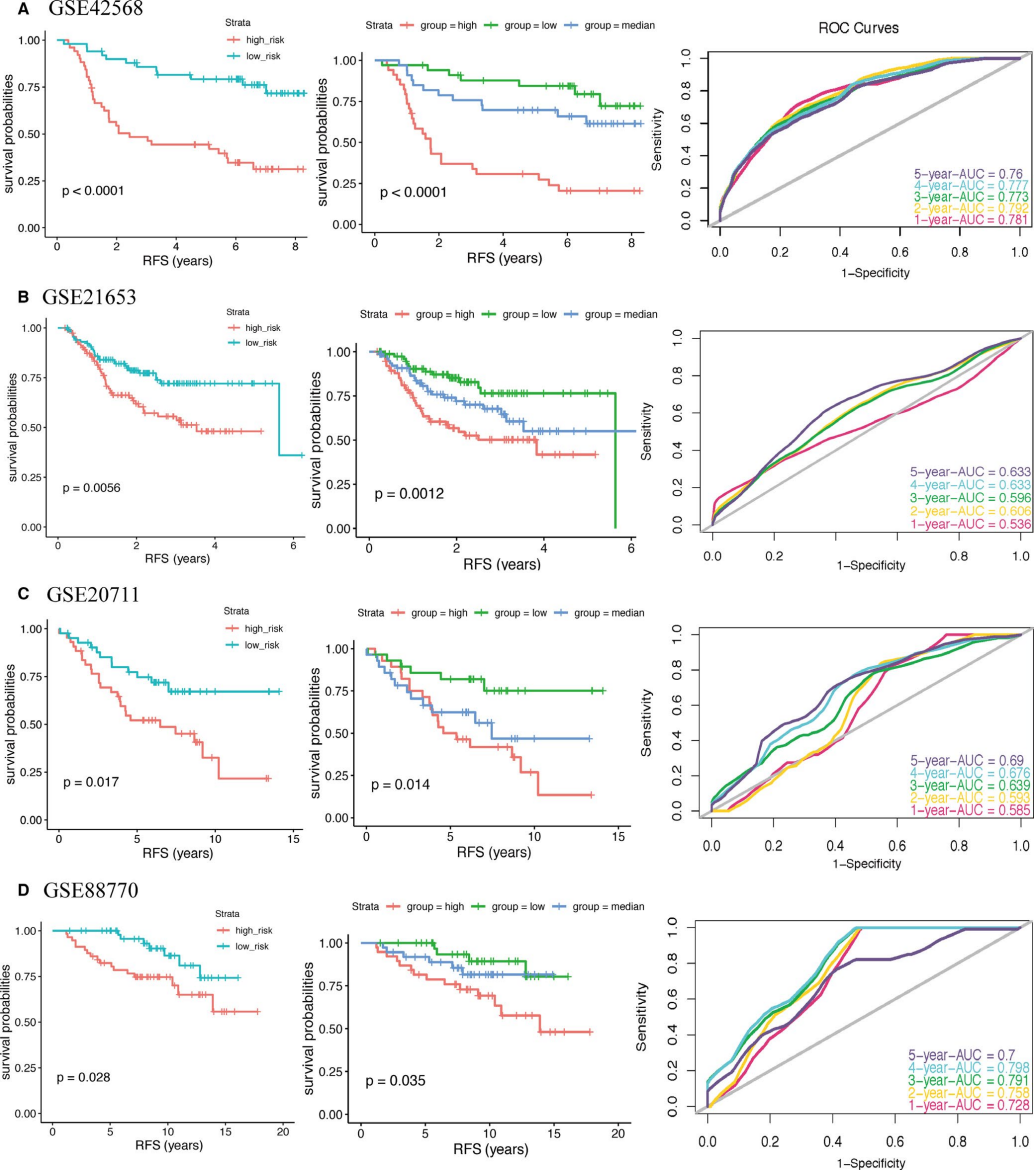
Kaplan‐Meier survival curve and ROC curves for prognostic signature in training set (A) and validation sets (B‐D). For ROC curves, we set the sensitivity to the ordinate and 1‐specificity to the abscissa. An AUC value close to 1.0 indicates a good diagnosis probability. The RFS of patients in high‐risk group was lower than that in low‐risk group. The ROC curves revealed a fair diagnostic property. *P*‐value < .05 (log‐rank tests) was considered statistically significant

Analysis of Kaplan‐Meier survival curve showed that low‐risk group had better RFS prognosis than patients included in the high‐risk group. Further analysis of the training set by time‐dependent ROC plots revealed that the immune‐related signature had outstanding diagnostic efficiency, with AUC values of 0.781, 0.792, 0.773, 0.777, and 0.760 for 1‐, 2‐, 3‐, 4‐, and 5‐year RFS (Figure 4A). In order to further confirm whether the immune‐related signature could be used as an independent prognostic factor, we performed a multivariate Cox proportional hazards regression analysis with clinical information of the patients, including T‐stage, lymph nodes, ER status, and grade. The results showed that, in addition to the immune‐related signature, ER status and lymph nodes could also be used as independent prognostic factors (Table 1).

### 3.4 Immune‐related prognostic signature validation

To evaluate the accuracy of the immune‐related signature, we analyzed three additional datasets (GSE21653, GSE20711, and GSE88770). Kaplan‐Meier curves confirmed that low‐risk group had better RFS than high‐risk group. Moreover the AUC values were 0.536, 0.606, 0.596, 0.633, and 0.633 in GSE21653 dataset; 0.585, 0.593, 0.639, 0.676, and 0.690 in GSE20711 dataset; 0.728, 0.758, 0.791, 0.798, and 0.700 in GSE88770 dataset, for 1‐, 2‐, 3‐, 4‐, and 5‐year RFS, respectively (Figure 4B‐D). Next, we performed multivariate Cox proportional hazards regression analysis on all datasets excluding GSE88770, which was not included in the analysis due to lack of T‐stage clinical information. The result further confirmed that the immune‐related signature is independent of other clinical parameters (Table 1).

### 3.5 Stratification analysis

**Figure 5:**
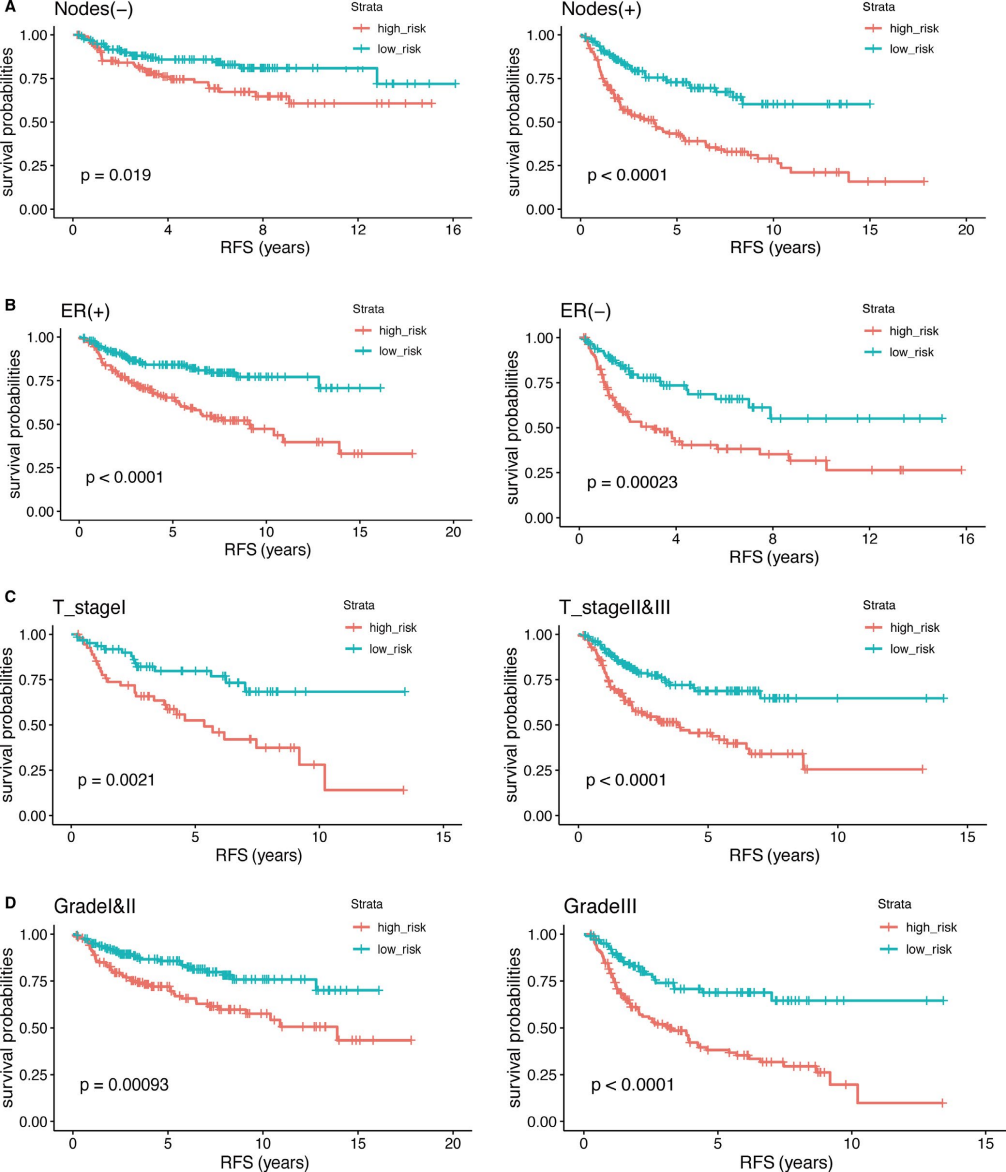
Kaplan‐Meier survival curve was drawn to predict the RFS of patients by stratification analysis of lymph nodes, ER status, T‐stage, and grade. The RFS of patients in high‐risk group was lower than that in low‐risk group. *P*‐value < .05 (log‐rank tests) was considered statistically significant

In the above‐mentioned multivariate Cox proportional hazards regression analysis, some clinical parameters were identified as independent prognostic factors. To confirm whether the immune‐related prognostic signature can be used to predict RFS of patients within the same clinical information subgroup, we performed a stratification analysis in training and validation sets. We divided the patients into different subgroup according to their clinical information. The results of the Kaplan‐Meier survival curve revealed that the low‐risk group had better RFS than high‐risk group in the same clinical subgroup (Figure 5).

### 3.6 Nomogram construction

**Figure 6:**
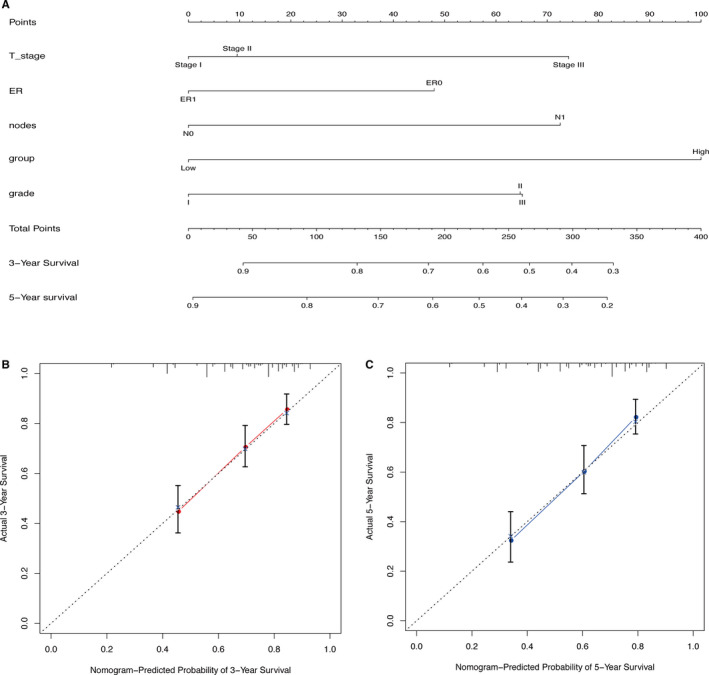
Nomogram constructed to predict the RFS of BC patients. A, BC RFS nomogram. To use the nomogram, each variable axis contains a value that should be matched to the each individual patient with a line upward to determine the number of points received for each variable value. The sum of these numbers is located on the total points axis, and a line should be drawn downward to the survival axis to determine the probability of a release event with three or five years. B‐C, Calibration curve for predicting RFS at 3‐year (B) and 5‐year (C) in all datasets. The nomogram‐predicted probability of RFS is plotted on the x‐axis; actual RFS is plotted on the y‐axis. The calibration curve showed that the nomogram had good prediction accuracy.

Next, we developed a nomogram including the immune‐related prognostic signature, as well as all the important independent clinical factors identified in the previous multivariate Cox regression analysis, to better quantitatively predict the 3‐year and 5‐year RFS events (Figure 6A). The cohort was composed of GSE42568, GSE21653, and GSE20711. (GSE88770 was not included in the analysis due to lack of T‐stage clinical information.) The calibration curves indicated that the nomogram had excellent performance (Figure 6B‐C).

### 3.7 Gene set enrichment analysis

**Figure 7:**
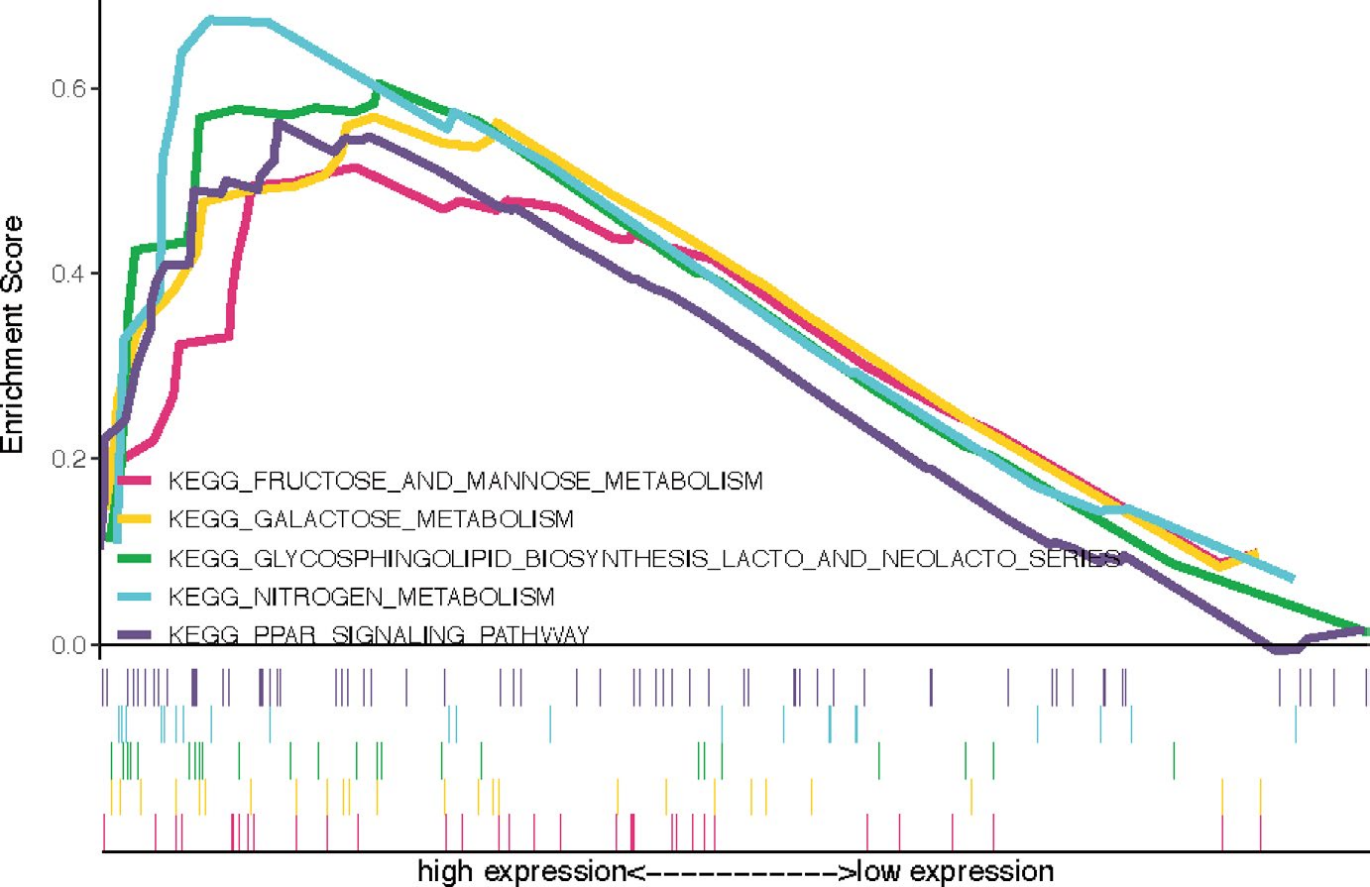
Gene set enrichment analysis between high‐ and low‐risk groups. The c2.cp.kegg.v6.2.symbols.gmt gene set was used as reference. The number of permutations was 1000. The maximum and minimum sizes for gene sets were set at 500 and 15, respectively. FDR is the adjusted P‐value after multiple hypothesis testing, FDR <25% (Benjamini‐Hochberg) was used as a cutoff to identify significant gene sets

We also performed a gene set enrichment analysis to better determine the expression differences between high‐ and low‐risk groups. FDR <25% was used as cut‐off criteria. The top five KEGG pathways enriched in high‐risk and low‐risk sample groups were fructose and mannose metabolism, galactose metabolism, glycosphingolipid biosynthesis lacto and neolacto‐series, nitrogen metabolism, and PPAR signaling pathway (Figure 7).

### 3.8 Experimental verification of the prognostic signature

**Figure 8:**
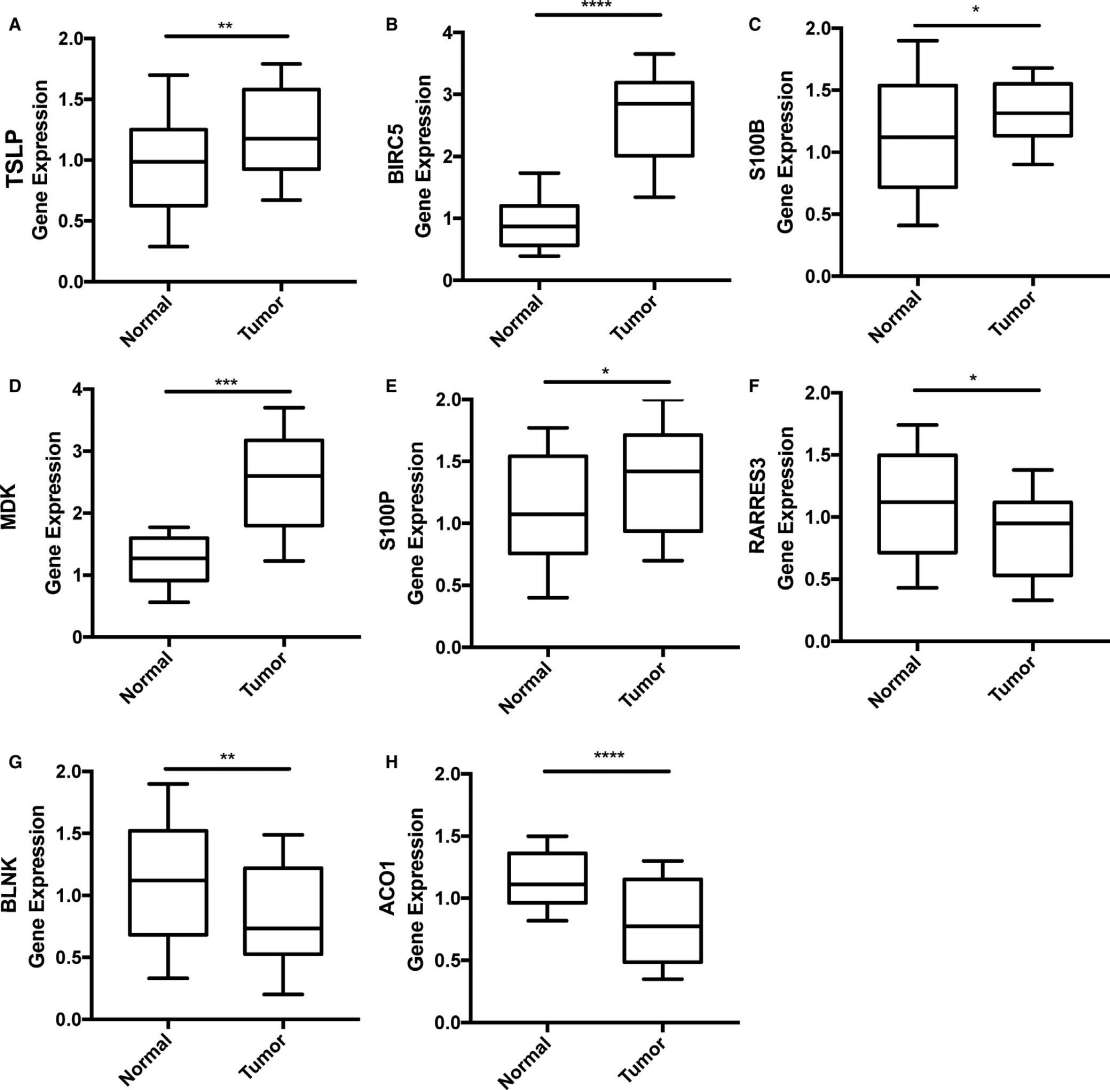
Experimental validation of the immune‐related prognostic signature in BC and adjacent tissues by qRT‐PCR. The expression of danger genes were upregulated in BC tissues, while the expression of protect genes were upregulated in adjacent tissues. *P*‐value < .05 (*t* test) was considered statistically significant. *P < .05; **P < .01; ***P < .001；****P < .0001. A. TSLP. B. BIRC5. C. S100B. D. MDK. E. S100P. F. RARRES3. G. BLNK. H. ACO1

To further verify the accuracy of the immune‐related prognostic signature, we evaluated the expression levels of *TSLP, BIRC5, S100B, MDK, S100P*, *RARRES3, BLNK,* and *ACO1* in 40 pairs of BC and adjacent tissues samples by qRT‐PCR. The experimental results showed that the mRNA expression levels of protect genes were significantly higher in adjacent samples compared to BC samples, while expression of danger genes presented the opposite trend (Figure 8).

## 4 DISCUSSION

The workflow of immune‐related gene expression and clinical information preprocessing, gene signature generation, and verification are displayed in Figure 1. In this study, we identified an immune‐related prognostic gene signature to predict the recurrence of BC. GSE42568 was used as the training set, GSE21653, GSE20711, and GSE88770 as the validation sets. FDR <0.05 (Benjamini‐Hochberg) and $\left|\log\text{FC}\right|>1$ were used as the inclusion criteria to screen for differentially expressed genes, a total of 134 IRGs and 33 TFs were identified. Univariate Cox regression analysis was used to determine the association between differentially expressed IRG levels and RFS in training set. Lasso‐penalized Cox regression and stepwise multivariate Cox analysis were performed next to narrow the list of IRGs. Finally, an immune‐related gene prognostic signature was developed (8 genes). We also constructed an interaction network of IRGs and TFs. The Kaplan‐Meier survival curve revealed that low‐risk patients had better prognosis than those identified within the high‐risk group. Moreover ROC and multiple Cox regression analysis indicated that the immune‐related prognosis signature has good diagnostic efficiency to identify relapse‐free events, representing an independent risk factor for BC. Stratification analysis further demonstrated that the prognostic signature can be used to predict the RFS of BC patients within the same clinical subgroup. We also developed a nomogram that integrates clinical features and the IRGs signature, which calibration plots indicated that had excellent performance, to support the clinical assessment of BC patients. To further validate the prognostic signature, we conducted additional qRT‐PCR analysis, which showed that the protect genes were upregulated in adjacent tissues, while the risk genes were up‐regulated in the BC samples. In summary, this study developed and validated an immune‐related prognostic signature that may provide guidance for the future diagnosis of BC recurrence.

The biological functions of the identified immune‐related prognostic genes have been previously reported. Thymic stromal lymphopoietin (TSLP) is a cytokine associated with type 2 immunity and is also associated with the progression of various cancers, including BC, pancreatic cancer, gastric cancer, cervical cancer, and myeloma.^25^, ^26^, ^27^, ^28^, ^29^ TSLP is upregulated in BC and promotes proliferation and lung metastasis by inducing Bcl‐2 expression.^30^ Baculoviral IAP repeat containing 5 (BIRC5) is at the crossroads of diverse cancer signaling networks and is a well‐known cancer treatment target.^31^ In the past 20 years, BIRC5‐related treatment approaches include inhibitors for BIRC5‐partner proteins, homodimerization inhibitors, gene transcription inhibitors, mRNA inhibitors, and immunotherapy.^32^ In particular, BIRC5 immunotherapy used in combination with standard therapies or with targeted precision drugs have shown great anti‐cancer potential.^33^, ^34^, ^35^ Recent studies have also shown that S100 Calcium Binding Protein B (S100B) is upregulated in BC, melanoma, ovarian cancer, and colon adenocarcinoma.^36^, ^37^, ^38^, ^39^ Moreover S100B has shown potential as monitoring indicator for ER‐positive BC, helping on the assessment of patients regarding to response to endocrine therapy.^36^ Midkine (MDK) is an heparin‐binding growth factor that is upregulated in various human malignancies and plays an important role in promoting growth, survival, and migration of cancer cells, as well as cancer angiogenesis and metastasis.^40^, ^41^ S100 Calcium Binding Protein P (S100P) is involved in the transendothelial migration of triple negative BC cells and is significantly associated with disease‐free survival.^42^ Use of anti‐S100P antibody combined with chemotherapy showed to effectively improve the survival of BC patients.^43^, ^44^ Retinoic acid receptor responder 3 (RARRES3) is a new tumor metastasis suppressor gene that is downregulated in BC and inhibits lung metastasis of BC.^45^, ^46^ B‐cell linker protein (BLNK) is a tumor suppressor involved in pre‐B‐cell leukemogenesis, which inhibits JAK3/STAT5 signaling by binding to JAK3.^47^ Cytoplasmic aconitate hydratase (ACO1) is a protein involved in cytoplasmic and mitochondrial metabolism that when downregulated leads to cell death, potentially representing a new therapeutic strategy for cancer treatment.^48^

Several previous studies have reported prognostic signatures for BC.^49^, ^50^, ^51^, ^52^, ^53^, ^54^ Compared with the models, our prognostic model in this article has following advantages. Firstly, our prognostic model was build using several statistical analysis tools to ensure its rigor and accuracy. We started by selecting differentially expressed genes, and then further screened out differentially expressed IRGs. Instead of using just univariate Cox and lasso regressions, we also used stepwise multivariate analysis to further narrow down the IRGs. Furthermore, we not only constructed a prognostic signature, but also constructed an interaction network between IRGs and TFs (Figure 2C), which may provide guidance for further analysis. We also evaluated the expression levels of eight IRGs in the prognostic signature through qRT‐PCR experiments, which further validates our bioinformatic results. Nevertheless, this study also has some limitations. Since only datasets from the GEO database were used in our analysis, it future studies should also cover cross‐platform databases such as TCGA, SEER, and GTEx. Additionally, as BRCA can have different pathogenesis processes and prognosis that are associated with diverse subtypes, it could be more accurate and meaningful to recognize an immune‐related prognosis for distinct BRCA subtypes.

## CONFLICT OF INTEREST

The authors claim no conflict of interest.

## AUTHOR'S CONTRIBUTION

ZT and JT wrote this article. GW revised this article. XL, QY, and YW conducted all statistical analyses.

## Supporting information

Fig S1

Fig S2

Table S1

## Data Availability

The data that support the findings of this study are available in Gene Expression Omnibus databases at https://www.ncbi.nlm.nih.gov/geo/query/acc.cgi, reference number: GSE42568, GSE21653, GSE20711 and GSE88770.
