# Supplementary figures and images for: An immune‐related prognostic signature for predicting breast cancer recurrence
Source: Cancer Med. 2020 Aug 25;9(20):7672–85. doi: 10.1002/cam4.3408 (PMC7571818; doi:10.1002/cam4.3408)

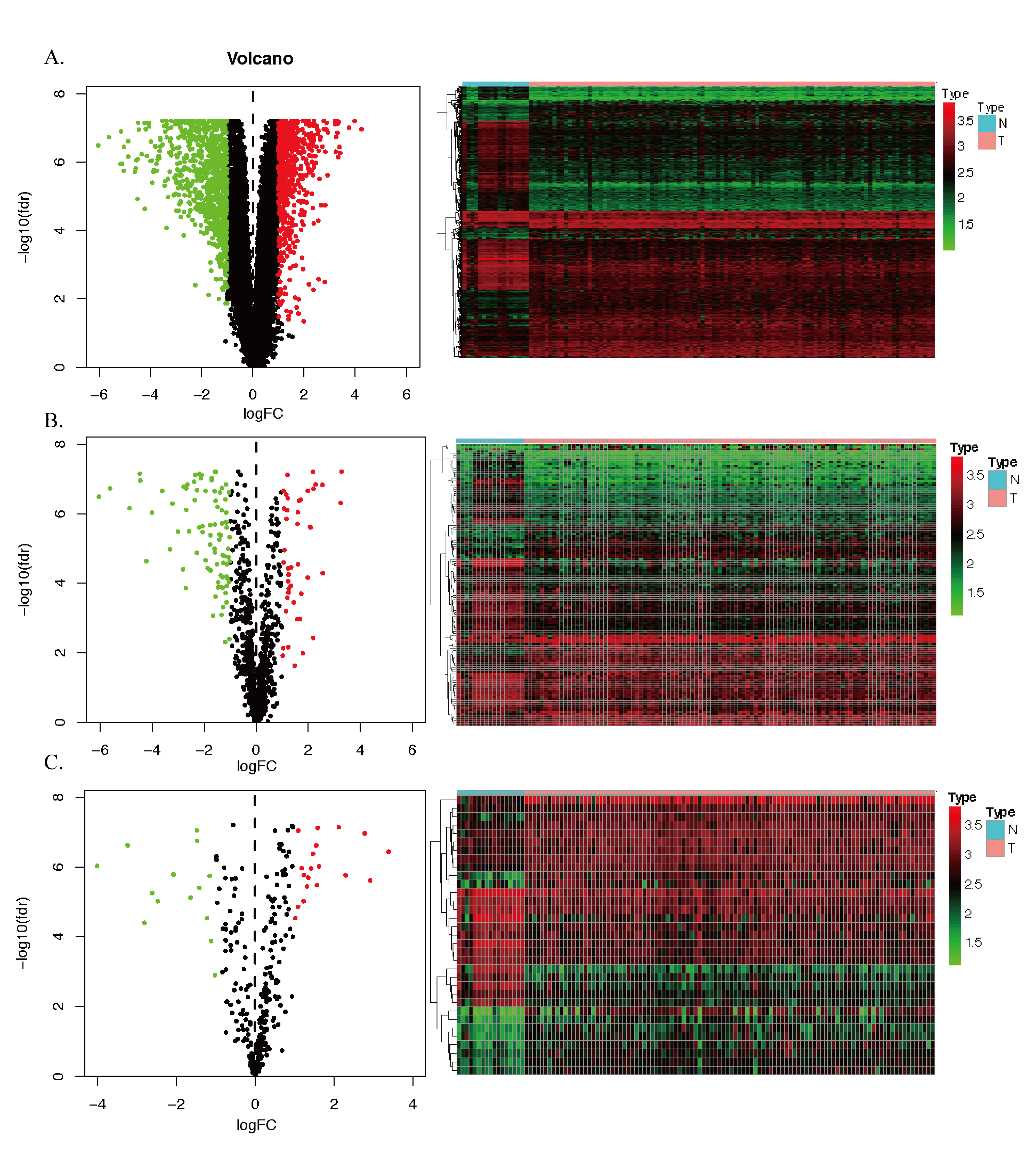

Supplement: Supplementary file 1 — Fig S1 [file CAM4-9-7672-s001.tif]

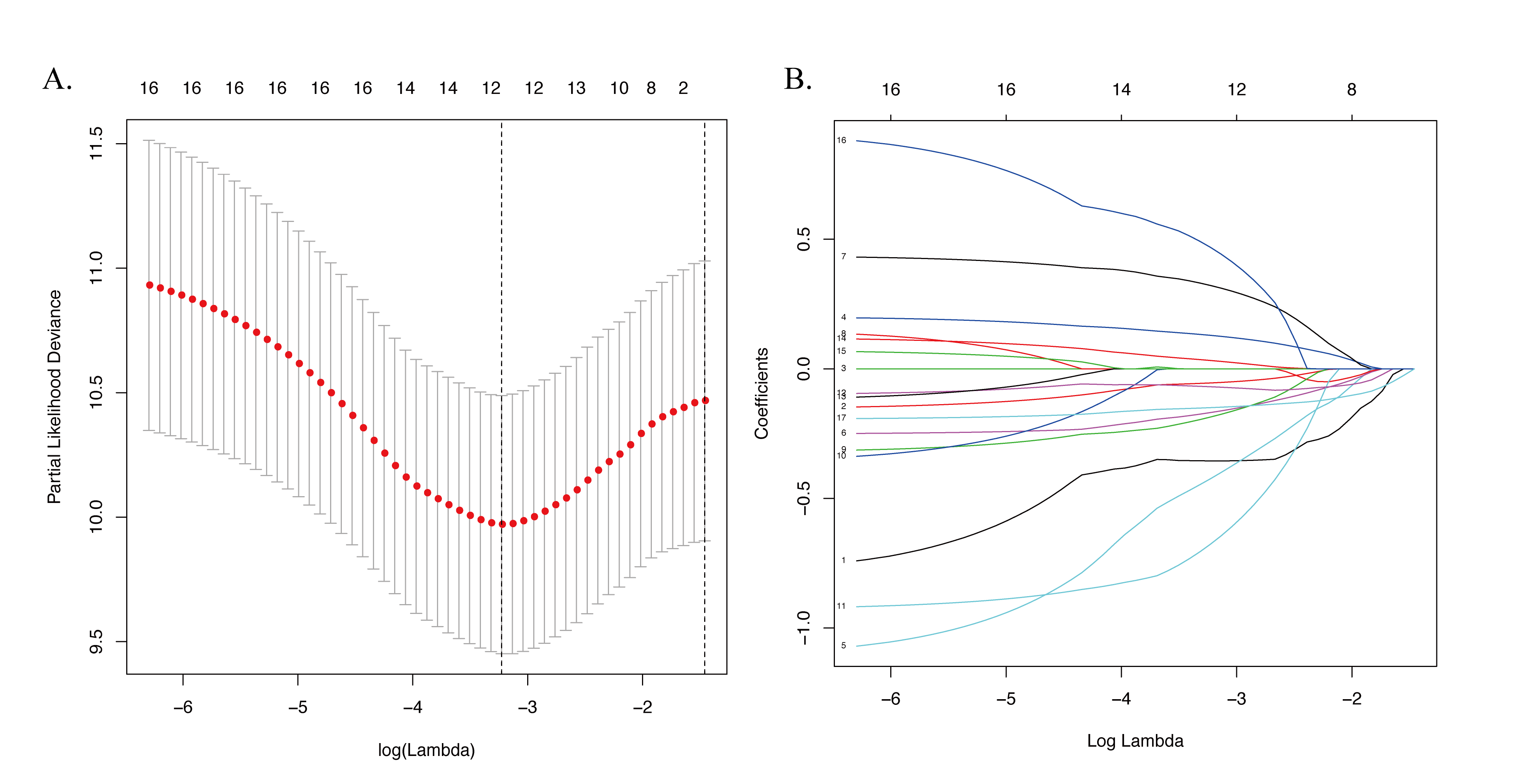

Supplement: Supplementary file 2 — Fig S2 [file CAM4-9-7672-s002.tif]
