# Supplementary material for: An immune‐related prognostic signature for predicting breast cancer recurrence
Source: Cancer Med. 2020 Aug 25;9(20):7672–85. doi: 10.1002/cam4.3408 (PMC7571818; doi:10.1002/cam4.3408)
Supplement: Supplementary file 3 — Table S1 [file CAM4-9-7672-s003.docx]

| Table S1. Primer sequences used to amplify target genes in human by real-time RT-PCR. | |
| --- | --- |
| Target Gene | Primer Sequence |
| TSLP |  |
| forward | 5′- ATGTTCGCCATGAAAACTAAGGC-3′ |
| reverse | 5′- GCGACGCCACAATCCTTGTA-3′ |
| BIRC5 |  |
| forward | 5′- AGGACCACCGCATCTCTACAT-3′ |
| reverse | 5′- AAGTCTGGCTCGTTCTCAGTG-3′ |
| S100B |  |
| forward | 5′-TGGCCCTCATCGACGTTTTC-3′ |
| reverse | 5′- ATGTTCAAAGAACTCGTGGCA-3′ |
| MDK |  |
| forward | 5′- CGCGGTCGCCAAAAAGAAAG-3′ |
| reverse | 5′-TACTTGCAGTCGGCTCCAAAC-3′ |
| S100P |  |
| forward | 5′- AAGGATGCCGTGGATAAATTGC-3′ |
| reverse | 5′- ACACGATGAACTCACTGAAGTC-3′ |
| RARRES3 |  |
| forward | 5′- GAGATTTTCCGCCTTGGCTAT-3′ |
| reverse | 5′- CCGGGGTACTCACTTGGAG-3′ |
| BLNK |  |
| forward | 5′- CCCGCCAGTCAGAAGTTGAG-3′ |
| reverse | 5′- AGTCCCTTCGAGGAACACTTG-3′ |
| ACO1 |  |
| forward | 5′-AACCCATTCGCACACCTTG-3′ |
| reverse | 5′-ATGGTAAGCGCCCATATCTTG-3′ |
| GAPDH |  |
| forward | 5′-GAAGGTGAAGGTCGGAGTC-3′ |
| reverse | 5′-GAAGATGGTGATGGGATTTC-3′ |
